# Gout and hyperuricaemia: modifiable cardiovascular risk factors?

**DOI:** 10.3389/fcvm.2023.1190069

**Published:** 2023-05-25

**Authors:** Michel Burnier

**Affiliations:** Faculty of Biology and Medicine, University of Lausanne, Switzerland and Hypertension Research Foundation, St Légier, Switzerland

**Keywords:** uric acid, hypertension, gout, cardiovascular mortality, tissue deposits, uric acid lowering treatments

## Abstract

Gout and hyperuricaemia are two clinical situations associated with an elevated risk of developing cardiovascular (heart failure, myocardial infarction, stroke) and metabolic and renal complications. One reason is probably related to the fact that the prevalence of hyperuricaemia and gout is high in clinical situations, which themselves involve a high cardiovascular risk, such as hypertension, diabetes, chronic kidney disease or obesity. However, recent studies suggest that hyperuricaemia may promote cardiovascular complications independently of other cardiovascular risk factors, by inducing chronic inflammation, oxidative stress, and endothelial dysfunction. The questions that arise today concern primarily the treatment of asymptomatic hyperuricaemia. Should it be treated to decrease the patients' cardiovascular risk and if so, starting from which level and towards which target? There are now several pieces of evidence indicating that this might be useful, but data from large studies are not unanimous. This review will discuss this issue as well as new well-tolerated treatments, such as febuxostat or SGLT2 inhibitors, which lower uric acid levels, prevent gout and lower the risk of cardio-renal events.

## Introduction

Hyperuricaemia and gout are highly prevalent in the population of developed countries particularly in men. Indeed, gout, considered for centuries to be a disease of rich, well-fed people, affects about 2%–3% of men and hyperuricaemia, defined in Europe as a uric acid blood level >360 μmol/L (408 μmol/L in the United States and 420 μmol/L in Japan (1, 2), is present in 25%–30% of men in Switzerland (3). The European and American recommendations concerning the treatment of hyperuricaemia and gout are clear today: patients with asymptomatic hyperuricaemia should not receive uric acid lowering treatments and should be treated with lifestyle corrections (2, 4, 5). By contrast, all patients who suffered an attack of gout should receive—in addition to non-pharmacological approaches—a specific treatment to lower their uric acid level below the crystallisation threshold of less than 360 *μ*mol/l and even below 300 μmol/l in the case of severe gout. Recommendations for patients with gout are not controversial. However, the attitude towards asymptomatic hyperuricaemia and its role in kidney and cardiovascular diseases are regularly questioned (6–8). The main reason for reconsidering the therapeutic abstention in asymptomatic patients is due to the fact that hyperuricaemia is frequently found to be an independent cardiovascular risk factor in epidemiological studies. Therefore, some national recommendations propose treating asymptomatic hyperuricaemia when levels exceed a certain threshold, e.g., > 480 μmol/L or > 500 μmol/L (i.e., > 8 or 9 mg/dl) (1) and others recommend to measure plasma uric acid levels as a cardiovascular risk factor in some patients groups such as patients with hypertension (9).

## Hyperuricaemia, gout, and cardiovascular risk

The first associations between the level of uric acid and the occurrence of cardiovascular events were reported in the 1950s already (10). In 1999, a first analysis of the Framingham Heart Study involving 6763 subjects with an average age of 47 years, suggested that baseline uric acid was predictive of the risk of cardiovascular mortality and coronary events in women but not in men (11). However, after correcting for other cardiovascular risk factors, this association was no longer significant, suggesting that uric acid was not the cause of the occurrence of cardiovascular events but rather a marker. In contrast, the first analysis of the National Health and Nutrition Examination Survey (NHANES I) in the United States, using data collected between 1971 and 1975 with a follow-up of 16 years, concluded that uric acid was an independent risk factor for cardiovascular events in men as well as in women, even after correcting for other known cardiovascular risk factors (12). Several more recent analyses from the NHANES program subsequently confirmed the link between uric acid and hypertension and the risk of global and cardiovascular mortality in diabetic patients (13, 14). Moreover, numerous analyses have been conducted in various populations to analyse the association between uric acid and the cardiovascular risk in greater details (15–20). In these studies, hyperuricaemia is described as a predictive factor, not only for hypertension and diabetes but also for heart disease, atrial fibrillation, myocardial infarction, heart failure, cerebrovascular events and chronic kidney disease. A systematic review and dose-response meta-analysis of over one million subjects has found a significant positive association between uric acid levels and the risk of cardiovascular mortality with a stronger association in women than men (21). Mendelian randomization studies have produced conflicting results regarding the causal implication of uric acid in the development of cardiovascular complications. Thus, Keenan et al. did not found any evidence supporting a causal role of circulating serum urate levels in type-2 diabetes, coronary heart disease, ischemic stroke, or heart failure (22). In contrast, Yang et al. reported some evidence for a causal effect of genetically determined serum urate level on heart failure (23). A similar causal effect was found for cardiovascular death and sudden cardiac death in 3,315 patients of the Ludwigshafen Risk and Cardiovascular Health Study (24). Similar results were found in the UK biobank but Mendelian Randomization analyses suggested a causal effect of hyperuricemia, but not gout, on cardiovascular diseases (25). In hypertension, the Mendelian randomization and clinical trial data from the UK Biobank tended to support an effect of higher serum urate on increasing blood pressure (26). Yet, as reviewed by Sanchez-Losada et al, though several studies were positive, the causal effect of elevated serum urate on blood pressure remains uncertain (27). Therefore, the causal character of the association between uric acid and cardiovascular events always remains a topic of debate because hyperuricaemia is very often associated with other risk factors such as dyslipidaemia, hypertension, obesity or diabetes. Hence, one cannot exclude an inverse causality or the effect of residual confounding factors, such as alcohol or excessive consumption of certain sugars. In this respect, in the Swiss investigation, conducted among a large group of general practitioners, the patients who presented with hyperuricaemia and/or gout were hypertensive in nearly 70% of cases and diabetic in ∼25% of cases (3). A quarter of these patients also had chronic kidney disease. Of note, in hyperuricemic adolescents with newly diagnosed hypertension, lowering uric acid has been reported to lower BP suggesting a pathogenic role of uric acid in the development of hypertension in some subjects (28). However, similar results were not always obtained in adults (27, 29). Most recent studies investigated the impact on cardiovascular risk of the trajectory of the uric acid level over several decades in younger populations. In this context, the CARDIA study showed that subjects (men and women) whose uric acid level increases the most over an average period of 10 years have a 2.89 fold increase in the risk of developing heart disease, heart failure, or a cerebrovascular events when compared with those whose uric acid level remains stable over time (30). As expected, hypertension, diabetes and obesity were much more frequent in subjects whose uric acid level increased over time.

## Why is hyperuricaemia related to an elevated cardiovascular risk?

**Figure 1:**
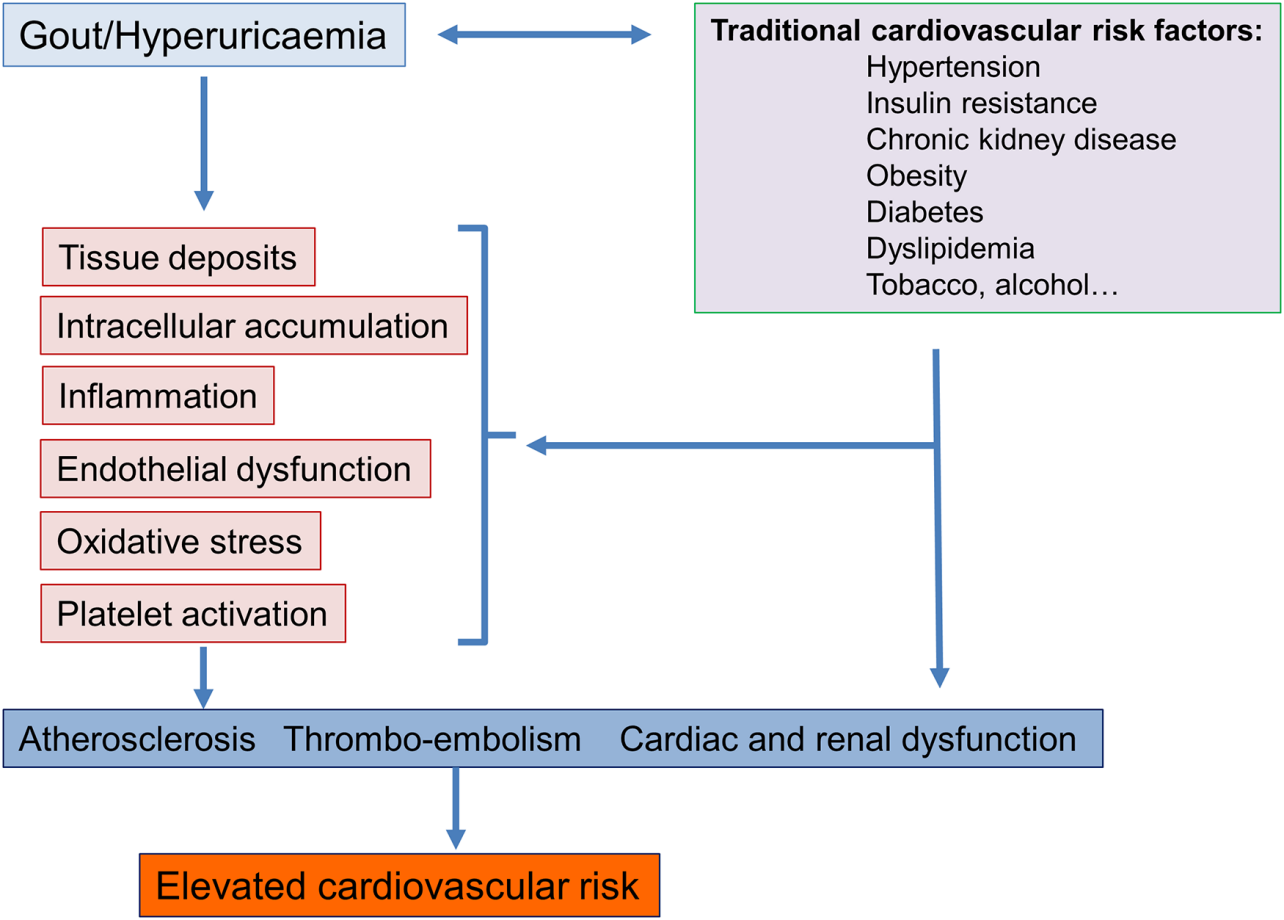
Potential mechanisms involved in the association between gout and hyperuricaemia and an elevated cardiovascular risk.

A priori, one could simply conclude that hyperuricaemia and gout are associated with a high cardiovascular risk because they develop in patients whose intrinsic cardiovascular risk is elevated, such as patients with hypertension, diabetes, renal failure, and ischaemic cardiomyopathy. However, looking at renal damages caused by hyperuricaemia, histological lesions are characterised by uric acid crystalline and also microcrystalline deposits associated with interstitial inflammation and renal fibrosis indicating that an excess of uric acid in itself causes tissue lesions. Today, numerous experimental studies have enable to better define the negative impact of hyperuricaemia and gout on the cardiovascular system (18, 19). As indicated in Figure 1, several mechanisms are involved, one of which is an accumulation of uric acid crystals in tissues (including in the heart and certain vessels (31, 32), the development of systemic inflammation with activation of neutrophils and macrophages and stimulation of pro-inflammatory cytokines associated with an inflammasome activation, endothelial dysfunction, and increase oxidative stress (33). These factors, some of which are responsible for joint disorders, are nowadays recognized as being implicated in the development of atherosclerosis and cardiovascular complications (34, 35). Another mechanism of toxicity of uric acid is the intracellular deposition of uric acid leading to elevated concentrations of uric acid within cells (36, 37). This may occur in the kidneys and in the liver. In this latter organ, intracellular accumulation of uric acid is thought to mediate oxidative stress to the mitochondria and has been associated with metabolic effects such as insulin resistance and hepatic fat accumulation and the development of atherosclerosis (37). As intracellular uric acid levels cannot be measured, the determination of plasma xanthine oxidase activity may be another diagnostic approach to identify patients in whom intracellular accumulation of uric acid may occur (38).

## Prevention of cardiovascular complications in gout and hyperuricaemia: which target?

As discussed above, the main therapeutic objective of the treatment of gout and hyperuricaemia is to decrease the plasma uric acid levels to below 360 μmol/L in order to prevent tissue deposits and gout attacks (39). Is this target adequate to prevent cardiovascular complications associated with hyperuricaemia? This is the question asked by a working group of the Italian Hypertension Society in its project entitled URRAH for “Uric acid Right for heArt Health project” (40). In this program, several threshold analyses were performed using data from the general population of Italy that involved 23,475 subjects with an average age of 57 ± 15 years, 49% of whom were women and whose average blood pressure was 143/85 ± 24/13 mmHg. The subjects were followed for nearly 20 years. As indicated in Table 1, the thresholds of the plasma uric acid levels associated with the highest risk of cardiovascular outcomes are all lower than the threshold actually recommended for the prevention of gout (40–43). These observations thus pose the question of future recommendations but these thresholds need to be validated in prospective randomised controlled studies. One possible explanation may be that some patients with an apparently “normal” serum uric acid develop cardiovascular complications due to the intracellular accumulation of uric acid. More recently, the same Italian authors proposed using the ratio of urinary uric acid/creatinine as a predictive factor of cardiovascular risk, a ratio greater than 5.35 having good predictive value of the cardiovascular risk associated with hyperuricaemia (44).

**Table 1:** Thresholds of plasma levels of uric acid associated with better prevention of events according to the URRAH (uric acid right for heart health) project of the Italian hypertension society.

Complication	Uric acid in mol/L	Uric acid in mg/dl	HR if value above this threshold
Total mortality	282	4.7	1.51 (95% CI 1.40–1.63) *p* < 0.001
CV mortality	336	5.6	1.59 (95% CI 1.43–1.76) *p* < 0.001
Fatal infarct	342	5.7 M: 5.49 W: 5.26	ROC curve parameter: AUC: 0.614 (95% CI 0.607–0.620)
Heart failure	320	5.34	1.46 (95% CI 1.17–1.83) *P* = 0.001
Fatal heart failure	293	4.89	1.59 (95% CI:1.23–2.05) *P* < 0.0001
Cerebrovascular accident	287	4.79	1.249 (95% CI 1.041–1.497) *p* = 0.016

Cox model for independent variables. HR, hazard ratio, CI, confidence interval.

AUC, area under the curve.

From references 40–43.

## Impact of uric acid lowering drugs on cardiovascular and renal complications

If hyperuricaemia and gout should play a significant role in the development of cardiovascular and renal outcomes, a decrease in uric acid levels brought about by xanthine oxidase inhibitors (allopurinol or febuxostat) or an inhibition of the inflammation caused by deposits of uric acid should be accompanied by a significant reduction of mortality and cardiovascular and renal events. Today the level of evidence of such protection from a reduction of uricemia remains low due to the heterogeneity of the results. Several cohort studies have suggested that treatment with allopurinol is associated with a reduction in cardiac events, such as myocardial infarction or heart failure (45–48). Patients on a high dose of allopurinol appear to have a lower incidence of cardiovascular complications than those on low doses (45). However, these findings have not always been confirmed (49). In a meta-analysis of 10 clinical studies with 738 participants systolic BP decreased by 3.3 mm Hg (95% confidence interval [CI], 1.4–5.3 mm Hg; *P* = .001) and diastolic BP decreased by 1.3 mm Hg (95% CI, 0.1–2.5 mm Hg; *P* = .03) in patients treated with allopurinol when compared with the control group (50).

A decrease in the incidence of cardiovascular outcomes has been reported with treatments that inhibit the inflammatory reaction in gout, such as colchicine and interleukin 1 inhibitors, but these treatments are not frequently used chronically (19). With regard to xanthine oxidase inhibitors (allopurinol, febuxostat), the ALL-HEARTstudy, a multicentre, prospective, randomised, open-label, blinded-endpoint trial, 5,937 patients with ischemic heart disease but no history of gout were randomly assigned to receive allopurinol or usual care (51). After a mean follow-up time of 4.8 years, there was no difference in the incidence of cardiovascular endpoints such as non-fatal myocardial infarction, non-fatal stroke, or cardiovascular death or in all-cause mortality. Based on these results, authors concluded that allopurinol should not be used for the secondary prevention of cardiovascular events in patients with ischaemic heart disease. More recent studies compared the cardiovascular effects of allopurinol and febuxostat in patients with a high cardiovascular risk. The first studies comparing two xanthine oxidase inhibitors [CARES (52), FAST (53)] did not show superiority of one compound over the other. In fact, a slight increase in the cardiovascular risk was observed on febuxostat, which was also seen in a large national monitoring study in Austria (54). However, this difference was not seen in the analysis of another large registry in Korea (55). Moreover, major flaws in the conduct of the CARES trial have been identified, which may relativize the difference in cardiovascular risk observed between allopurinol and febuxostat (56). In the FREED (Febuxostat for Cerebral and CaRdiorenovascular Events PrEvEntion StuDy) study, which included elderly patients with hyperuricaemia and an elevated risk of cerebral, cardiovascular or renal complications, patients were randomised to receive either febuxostat for 3 years or a conventional treatment (57). The use of febuxostat was associated with a significant decrease in the primary objective, which comprised 3 elements (cerebral, cardiac and renal events) (23.3% vs. 28.7%, *p* = 0.02). However, the statistical significance was essentially due to a decrease in the risk of albuminuria (58). More recently, the analysis of the largest cohort allowing allopurinol to be compared to febuxostat did not demonstrate any difference between the two treatments in terms of cardiovascular events. Nonetheless, the risk of total mortality was 16% lower in the febuxostat group (59).

Among the new drugs that have an effect on uric acid, sodium-glucose cotransporter 2 (SGLT2) inhibitors should be mentioned since they have a well-documented uricosuric effect. In a meta-analysis of 62 studies, SGLT2 inhibitors reduced circulating uric acid by 0.63 mg/dl (95% CI 0.59–0.61; 38 *μ*mol/L, 95% CI 41–35) (60). In another epidemiological study using the Danish nationwide health registries, the three year risk of gout was assessed in 11,047 pairs of matched SGLT2 inhibitors and glucagon-like peptide-1 receptor agonists (GLP1-RA) users (61). The incidence rate of gout was significantly lower in patients initiated with a SGLT2 inhibitors with a hazard ratio of 0.58 (0.44 to 0.75).The current hypothesis on the mechanisms whereby SGLT2 inhibitors lower serum uric acid is that glucose promotes urinary uric acid excretion through the high urine flow rate induced by the glycosuria. SGLT2-inhibitors-induced uricosuria is mediated by cellular mechanisms involving the transporters URAT1 and the suppression of the activity of GLUT9 transporting both glucose and uric acid (62). The SIRTUIN pathway may also be involved by reducing xanthine oxidase activity and hence serum uric acid levels. In addition, several studies have recently demonstrated that the SGLT2 inhibitors decrease the risk of gout in patients with type 2 diabetes (63–66, 67). The contribution of the reduction in uric acid levels to the observed cardiovascular and renal protection induced by SGLT2 inhibitors is now being evaluated (68).

With regard to renal protection by xanthine oxidase inhibitors, several reviews have concluded that there is insufficient evidence to support the renoprotective effects of urate-lowering agents in CKD patients with hyperuricemia, but some specific patients groups may benefit from lowering their uric acid levels (6, 7, 69–71). Three prospective, randomised, placebo-controlled studies (FEATHER (72), PERL and CKD-FIX (73) were conducted to demonstrate the ability of these drugs to slow the worsening of renal function in patients with stage 3 chronic renal failure, one of which involved patients with type 1 diabetes (74). These three studies did not demonstrate a significant effect of the inhibition of xanthine oxidase on worsening of renal function. However, it is possible that they included patients with renal stages that were too advanced to be slowed down. To support this hypothesis, a post-hoc analysis of the FEATHER trial has shown that febuxostat retarded the decline in kidney function among stage 3 CKD patients with asymptomatic hyperuricemia without proteinuria (75). Earlier treatment in CKD patients should therefore be studied in more detail.

## Conclusions

Asymptomatic hyperuricaemia and gout are two clinical conditions associated with a high risk of cardiovascular events and mortality. One of the main reasons is that hyperuricaemia develops mainly in patients at high cardiovascular risk. Nonetheless, there is growing experimental evidence suggesting that hyperuricaemia contributes to the progression of cardiovascular pathologies through tissue deposits and intracellular uric acid accumulation leading to a chronic inflammation, particularly in patients with gout. Today, it is not recommended to treat asymptomatic hyperuricaemia, unless the uric acid level is very elevated. Recently, the safety of reaching lower uric acid levels (independently of the drug class used) has been questioned in line with the U-shaped association of urate with mortality in some observational studies (76). Nonetheless, measurement of the uric acid level is now called for in several recommendations of international hypertension societies to optimise management of cardiovascular risk factors (9, 77). Current data suggest that gout and hyperuricaemia are modifiable cardiovascular risk factors, but to prevent the development of cardiovascular complications, it is possible that one must reach uric acid thresholds that are lower than those currently recommended as suggested by Italian researchers, but this remains to be demonstrated in randomised prospective studies. To reach these targets, xanthine oxidase inhibitors remain the first-line treatment, but treatment persistence is often low (78). In the long-term management of hyperuricaemia and the prevention of gout attacks, febuxostat is as effective as allopurinol but with better tolerance and persistence of the treatment (78) which allows a larger number of patients to be correctly treated. The place of new therapeutic approaches such as SGLT2 inhibition remains to be precised. Today, there is a consensus to further investigate the role of uric acid in the development of renal and cardio-metabolic complications through newly designed clinical trials using all new technologies (38, 79) to test the real benefits of lowering uric acid in hyperuricemic subjects.
